# Nurse Competencies, Patient Characteristics, and Pain Management Protocol Adherence in Intensive Care Units: A Multicenter Cross‐Sectional Study Accounting for ICU Clustering

**DOI:** 10.1155/jonm/7066240

**Published:** 2026-10-28

**Authors:** Yasaman Shafie Motlagh Kashani, Mahboubeh Shali, Maryam Esmaieli, Shima Haghani

**Affiliations:** ^1^ Department of Critical Care Nursing School of Nursing and Midwifery Tehran University of Medical Sciences Tehran Iran tums.ac.ir; ^2^ Department of Biostatistics Iran University of Medical Sciences Tehran Iran iums.ac.ir

**Keywords:** clinical competence, intensive care units, pain management, patient characteristics, self-efficacy

## Abstract

**Aim(s):**

To examine the associations between nurse competencies (knowledge, self‐efficacy, and attitude), patient characteristics, and pain management protocol adherence in adult intensive care units, while accounting for between‐ICU clustering.

**Background:**

Adherence to pain management protocols remains suboptimal despite available guidelines. Nurse competencies are essential for evidence‐based practice, yet their translation into clinical performance may depend on contextual factors. ICU‐level organizational context has been described in recent reviews, but empirical examination of these relationships remains limited.

**Method(s):**

This multicenter cross‐sectional study included 525 ICU nurses from seven hospitals in Tehran, Iran (July–October 2025). One index patient was assessed for each nurse. ICU‐level organizational characteristics were assessed through unit observation and head nurse interviews. The intraclass correlation coefficient (ICC) for protocol adherence was computed from an empty mixed model. Multivariable linear regression with ICU entered as a fixed effect was used to identify individual‐level factors associated with adherence. Organizational context was retained as a descriptive variable only.

**Results:**

Protocol adherence was moderate (mean 8.5/12; 32.4% good adherence). The ICC for protocol adherence was 0.02 (95% CI: 0.001–0.15), indicating minimal clustering. In the multivariable model with ICU as a fixed effect (18 predictors), knowledge (*β* = 0.311), self‐efficacy (*β* = 0.290), and attitude (*β* = 0.276) remained independently associated with adherence (all *p* < 0.001). Patient characteristics remained significantly associated: substance use history (*β* = −0.138, *p* = 0.016), inability to communicate (*β* = −0.148, *p* = 0.010), GCS < 9 (*β* = −0.160, *p* = 0.008), and mechanical ventilation (*β* = −0.121, *p* = 0.028). The full model explained 53.4% of the variance in adherence. Organizational support across the seven ICUs ranged from 1 to 5 (weighted mean 3.42 ± 1.15).

**Conclusion(s):**

Nurse competencies and patient characteristics are independently associated with pain management protocol adherence in this multicenter ICU sample. Between‐ICU clustering was minimal (ICC = 0.02), suggesting that individual‐level factors are the primary correlates of adherence in this sample. Given the small number of ICU clusters (*n* = 7), organizational findings are descriptive only and require confirmation in larger multilevel studies.

**Implications for Nursing Management:**

Nurse managers may consider competency‐based pain management education and regular audits with feedback. Given the small number of ICU clusters (*n* = 7), these recommendations are hypothesis‐generating and require validation in larger multilevel studies.

## 1. Introduction

Pain is a prevalent and clinically significant problem among critically ill patients in intensive care units (ICUs), where invasive procedures, mechanical ventilation, and underlying illness frequently contribute to pain [1]. Effective pain management is an essential component of high‐quality ICU nursing care [2]. Although evidence‐based guidelines and standardized protocols provide clear recommendations for pain assessment, treatment, and reassessment [3], adherence to these protocols remains inconsistent across ICU settings [4, 5]. This persistent gap between recommended and actual practice underscores the need to understand the factors that influence nurses’ adherence to pain management protocols [6].

Nurse competencies—particularly knowledge, self‐efficacy, and attitude—are recognized as important determinants of pain management practice [7, 8]. Knowledge provides the foundation for evidence‐based care; self‐efficacy reflects confidence in one’s ability to perform specific clinical actions; and attitude represents the value placed on pain management and motivation to prioritize it [9, 10]. However, evidence suggests that adequate knowledge and positive attitudes do not necessarily translate into consistent protocol adherence [11]. This indicates that individual competencies alone may be insufficient to explain clinical performance.

Contextual factors may play a role in enabling or constraining the translation of individual competencies into clinical practice. Factors such as workload, staffing adequacy, resource availability, educational opportunities, and access to protocols can either facilitate or impede the implementation of evidence‐based care [12, 13]. The capability–opportunity–motivation–behavior (COM‐B) model proposes that behavior results from the interaction between individual capability and motivation with opportunities provided by the environment [14]. Recent reviews have described ICU pain‐management competency [7, 11]; however, these reviews are primarily descriptive, and empirical examination of whether contextual factors are associated with the competency–adherence relationship remains limited. More recent 2026 integrative and scoping reviews have further consolidated evidence on the role of organizational context in shaping ICU pain‐management competency, yet empirical examination of whether these contextual factors are associated with the competency–adherence relationship remains limited. Therefore, this study aimed to empirically examine the associations between nurse competencies, patient characteristics, and pain management protocol adherence in adult ICUs, while accounting for between‐ICU clustering.

## 2. Materials and Methods

### 2.1. Study Design and Setting

This multicenter cross‐sectional study was conducted between July and October 2025 in the ICUs of seven hospitals affiliated with Tehran University of Medical Sciences, Tehran, Iran. Each participating hospital contributed one ICU to the study. The participating hospitals included Imam Khomeini, Valiasr, Cancer Institute, Ziaian, Baharloo, Shariati, and Sina hospitals, representing general, specialized, trauma, and teaching facilities. The study was reported according to the Strengthening the Reporting of Observational Studies in Epidemiology (STROBE) guidelines for observational studies (see Supporting File 1 for completed checklist).

### 2.2. Participants and Sampling

The target population comprised all nurses working in the participating ICUs. Eligible nurses had at least 1 year of ICU experience, were actively employed during the study period, had direct patient care responsibility, and were willing to participate. Nurses who withdrew or completed less than 70% of questionnaires were excluded. A census sampling approach was used; all eligible nurses were invited to participate. Of 612 eligible nurses, 525 (85.8%) completed the study. A total of 87 nurses were excluded: 52 (8.5%) completed less than 70% of the questionnaires, and 35 (5.7%) withdrew or had incomplete data. The final analytic sample comprised 525 nurse–patient pairs.

For each participating nurse, one index patient was selected. The index patient was defined as the patient under the direct care of the participating nurse during the observed shift who met the following criteria: (1) adult (≥ 18 years), (2) admitted to the ICU for at least 24 h, and (3) not expected to be discharged or transferred during the observation period. If a nurse was caring for more than one eligible patient, the patient with the longest ICU stay was selected as the index patient to maximize observation time. To ensure that each patient was unique to one nurse, patient medical record numbers were checked against a precompiled exclusion list updated daily by the research team. Each index patient was unique to one participating nurse; no patient contributed observations for multiple nurses.

All eligible nurses were invited using a census sampling approach; therefore, the sample size was determined by the accessible study population rather than an a priori individual‐level power calculation.

### 2.3. Ethical Considerations

Written informed consent was obtained from all nurse participants. For patients able to consent, written informed consent was obtained directly. For patients unable to consent, consent was obtained from a legally authorized representative. Ethical approval was obtained from the Joint Ethics Committee of the School of Nursing and Midwifery and the School of Rehabilitation, Tehran University of Medical Sciences (IR.TUMS.FNM.REC.1404.023), on June 15, 2025. Institutional permission was obtained from the research deputy of each of the seven participating hospitals prior to data collection. Data collection began after ethics approval and institutional permissions were obtained.

### 2.4. Variables and Measurement

#### 2.4.1. Pain Management Protocol Adherence (Dependent Variable)

Adherence was assessed using a checklist developed based on the Iranian Ministry of Health pain management protocol (Resolution No. 586/139/d, June 9, 2024) [15]. The checklist was originally developed based on 14 binary items covering three domains: pain assessment/diagnosis (7 items), pain relief interventions (4 items), and pain reassessment (3 items). During finalization of the instrument, two pairs of items were combined into single composite items: (1) the items for “nonopioid analgesic for mild pain” and “weak opioid for moderate pain” were merged, and (2) the items for “reassessment timing” and “documentation/communication” were merged. Consequently, the final checklist contained 12 independently scored binary items across three domains (7 assessment + 3 intervention + 2 reassessment = 12 items), yielding a total score range of 0–12. Each item was scored as “yes” (performed) or “no” (not performed). A total score ≥ 10 was prespecified based on the Iranian Ministry of Health protocol threshold to indicate good adherence. The checklist was completed by the researcher through direct observation of nursing care and review of patient documentation.

#### 2.4.2. Nurse Competencies (Independent Variables, Individual Level)

Knowledge was assessed using the 31‐item McMillan et al. questionnaire [16], with each correct answer scoring one point (range 0–31). Self‐efficacy was assessed using the 17‐item Pain Management Self‐Efficacy Questionnaire [17], Persian version validated by Rezaei et al. [18], on a six‐point Likert scale (range 0–85). Attitude was assessed using the 20‐item McMillan et al. questionnaire [16] on a four‐point Likert scale (range 20–80).

#### 2.4.3. ICU‐Level Organizational Characteristics

Organizational characteristics were assessed at the ICU level (*n* = 7 ICUs) using a researcher‐developed questionnaire through direct unit observation and consultation with head nurses. Key variables included Nursing Activities Score (NAS) as a measure of ICU‐level workload [19]; availability of analgesics, pain management protocols, and patient‐controlled analgesia (PCA) pumps; presence of anesthesiology residents; availability of educational programs; documentation monitoring; and nonpharmacological pain management facilities. An organizational support composite score was created by summing three favorable ICU‐level characteristics: (1) educational programs (0–1), (2) resource availability (0–3), and (3) documentation monitoring (0–1), yielding a total range of 0–5. Content validity of the researcher‐developed questionnaire was confirmed by 12 experts (CVR: 0.81–0.87; CVI: 0.83–0.89). Internal consistency of the derived composite was not calculated because the ICU‐level sample (*n* = 7) is insufficient for reliable reliability estimation; this is acknowledged in the Limitations.

#### 2.4.4. Covariates

All prespecified covariates were retained in the final model regardless of statistical significance. Nurse covariates included age, educational level, work experience, ICU experience, and previous pain management training. Patient covariates included age, Glasgow Coma Scale (GCS) score, mechanical ventilation status, substance use history, and communication ability. Nurse sex, patient sex, and reason for admission were excluded a priori because they were redundant with other covariates or had insufficient variability (e.g., 78.5% of nurses were female).

### 2.5. Data Collection Procedure

After ethical approval and institutional permissions, the researcher identified eligible nurses during each study shift and explained the study objectives. After obtaining written informed consent, nurses completed questionnaires during rest periods. The researcher simultaneously assessed protocol adherence through observation and chart review, collected patient data from records, and assessed ICU‐level organizational characteristics through unit observation and head nurse interviews. Data collection continued across all shifts (morning, evening, and night) between July and October 2025.

### 2.6. Statistical Analysis

Analyses were performed using SPSS Version 26 (IBM Corp., Armonk, NY, USA). Descriptive statistics (frequencies, percentages, means, and standard deviations) were calculated. Normality was confirmed using the Kolmogorov–Smirnov test (*p* > 0.05). Pearson correlations examined bivariate associations.

The intraclass correlation coefficient (ICC) for protocol adherence across the seven ICUs was computed from an empty mixed model (random intercept only, no predictors) using the variance components procedure in SPSS, with ICU as the grouping variable. The ICC was 0.02 (95% CI: 0.001–0.15), indicating minimal clustering.

Because ICU‐level organizational support was collinear with the ICU fixed effect (organizational support is a group‐level variable assigned to individuals and correlates strongly with ICU membership), and the ICC was low, ICU was entered as a fixed effect in the multivariable regression model primarily to adjust for any residual between‐ICU differences. Organizational support was retained as a descriptive variable only, and no *p* value is reported for it.

The multivariable model contained 18 predictors: 12 individual‐level predictors (knowledge, self‐efficacy, attitude, nurse age, nurse education, nurse work experience, ICU experience, previous pain training, patient age, GCS, mechanical ventilation, substance use, and communication ability) plus 6 ICU dummy variables (with one ICU as reference). The final model was *F* (18, 506) = 32.15, *p* < 0.001, *R* ^2^ = 0.534, adjusted *R* ^2^ = 0.517.

Multivariable linear regression with ICU as a fixed effect was used to identify individual‐level factors associated with protocol adherence. All continuous predictors were mean‐centered. Multicollinearity was assessed using variance inflation factor (VIF) and tolerance (VIF < 10, tolerance > 0.1). Assumptions of linearity, homoscedasticity, and normality of residuals were verified.

## 3. Results

### 3.1. Participant Characteristics

A total of 525 ICU nurses participated. One index patient was assessed for each nurse. Mean nurse age was 34.6 ± 7.2 years; 78.5% were female, and 88.0% held a bachelor’s degree. Mean nursing and ICU experience were 9.8 ± 4.6 and 6.4 ± 3.8 years, respectively. Only 36.0% reported previous pain management training. Patient characteristics showed that 18.7% had substance use history, 40.6% were unable to communicate verbally, 32.0% had GCS < 9, and 46.7% were mechanically ventilated (Table 1).

**TABLE 1:** Characteristics of participating nurses and index patients (*N* = 525 nurse–patient pairs).

Nurse characteristics	Value
Age, years, mean ± SD	34.6 ± 7.2
Female, *n* (%)	412 (78.5)
Bachelor’s degree, *n* (%)	462 (88.0)
Married, *n* (%)	325 (61.9)
Nursing experience, years, mean ± SD	9.8 ± 4.6
ICU experience, years, mean ± SD	6.4 ± 3.8
Previous pain training, *n* (%)	189 (36.0)

**Patient characteristics**	***n* (%)**

Substance use history	98 (18.7)
Unable to communicate	213 (40.6)
GCS < 9	168 (32.0)
Mechanical ventilation	245 (46.7)

### 3.2. Descriptive Statistics of Main Variables

The mean protocol adherence score was 8.5 ± 2.3 out of 12, representing 70.8% of the maximum; 32.4% achieved good adherence (score ≥ 10). Mean scores for knowledge, self‐efficacy, and attitude were 18.3 ± 4.7, 52.7 ± 12.4, and 56.8 ± 9.1, respectively. ICU‐level organizational support had a weighted mean of 3.42 ± 1.15 (possible range 0–5; observed range 1–5). The mean NAS across the seven ICUs was 81.6 ± 7.4% (between‐ICU SD), ranging from 72% to 95% (Table 2; see Supporting Table S1 for per‐ICU values).

**TABLE 2:** Descriptive statistics of main study variables.

Variable	Mean ± SD	Possible range	% of maximum
Protocol adherence	8.5 ± 2.3	0–12	70.8
Knowledge	18.3 ± 4.7	0–31	59.0
Self‐efficacy	52.7 ± 12.4	0–85	62.0
Attitude	56.8 ± 9.1	20–80	71.0
ICU‐level organizational support	3.42 ± 1.15	0–5	68.4
NAS (%)	81.6 ± 7.4	72–95	—

*Note:* ICU‐level organizational support is reported as a weighted mean across the 525 nurses. NAS is reported as the mean of the seven ICU‐level values with between‐ICU SD.

### 3.3. Correlations Among Study Variables

All three competencies were positively correlated with protocol adherence: knowledge (*r* = 0.15, *p* < 0.001), self‐efficacy (*r* = 0.14, *p* = 0.001), and attitude (*r* = 0.13, *p* = 0.003). ICU‐level organizational support was also correlated with adherence (*r* = 0.15), but because organizational support is an ICU‐level variable, no *p* value is reported for this correlation. Competencies were intercorrelated (*r* = 0.46–0.53, all *p* < 0.001) (Table 3).

**TABLE 3:** Pearson correlations among main study variables.

Variable	1	2	3	4	5
1. Protocol adherence	1.00				
2. Knowledge	0.15^∗∗∗^	1.00			
3. Self‐efficacy	0.14^∗∗^	0.49^∗∗∗^	1.00		
4. Attitude	0.13^∗∗^	0.46^∗∗∗^	0.53^∗∗∗^	1.00	
5. ICU‐level organizational support	0.15^†^	0.12^†^	0.11^†^	0.10^†^	1.00

^†^ *p* value not reported because of cluster‐level dependence (*n* = 7 ICUs).

^∗∗^ *p* < 0.01.

^∗∗∗^ *p* < 0.001.

### 3.4. Multivariable Regression: Individual‐Level Factors With ICU as Fixed Effect

The ICC for protocol adherence was 0.02 (95% CI: 0.001–0.15), indicating minimal clustering. In the multivariable regression model with ICU as a fixed effect (18 predictors: 12 individual‐level + 6 ICU dummies), knowledge (*β* = 0.311, *B* = 0.153, *p* < 0.001), self‐efficacy (*β* = 0.290, *B* = 0.054, *p* < 0.001), and attitude (*β* = 0.276, *B* = 0.070, *p* < 0.001) were independently associated with protocol adherence. Patient characteristics remained significantly associated: substance use history (*β* = −0.138, *B* = −0.818, *p* = 0.016), inability to communicate (*β* = −0.148, *B* = −0.696, *p* = 0.010), GCS < 9 (*β* = −0.160, *B* = −0.784, *p* = 0.008), and mechanical ventilation (*β* = −0.121, *B* = −0.558, *p* = 0.028). Nurse age, education, work experience, ICU experience, previous pain training, and patient age were not significant predictors (Table 4).

**TABLE 4:** Multivariable regression analysis of individual‐level factors associated with protocol adherence (with ICU as fixed effect).

Predictor	*B*	SE	*β*	*t*	*p*	95% CI
(Constant)	9.15	0.65	—	14.08	< 0.001	7.87–10.43
Knowledge	0.153	0.019	0.311	8.05	< 0.001	0.116–0.190
Self‐efficacy	0.054	0.007	0.290	7.71	< 0.001	0.040–0.068
Attitude	0.070	0.010	0.276	7.00	< 0.001	0.050–0.090
Substance use history	−0.818	0.335	−0.138	−2.44	0.016	−1.476 to −0.160
Inability to communicate	−0.696	0.267	−0.148	−2.61	0.010	−1.221 to −0.171
GCS < 9	−0.784	0.288	−0.160	−2.72	0.008	−1.350 to −0.218
Mechanical ventilation	−0.558	0.250	−0.121	−2.23	0.028	−1.049 to −0.067
Nurse age	0.011	0.014	0.036	0.81	0.418	−0.016–0.038
Education	0.190	0.200	0.027	0.95	0.342	−0.203–0.583
Work experience	0.014	0.020	0.029	0.71	0.478	−0.025–0.053
ICU experience	0.017	0.021	0.027	0.83	0.407	−0.024–0.058
Previous pain training	0.145	0.198	0.030	0.73	0.465	−0.244–0.534
Patient age	0.008	0.012	0.025	0.67	0.503	−0.016–0.032

*Note: β* coefficients were computed as *B* × (SD_predictor/SD_outcome). SD_outcome = 2.3. SD_previous_pain_training = 0.48 (binary). ICU‐level organizational support was collinear with the ICU fixed effect and could not be included in the model; it is retained as a descriptive variable only (weighted mean 3.42 ± 1.15). *R* ^2^ = 0.534; adjusted *R* ^2^ = 0.517; *F* (18, 506) = 32.15; *p* < 0.001. ICU fixed effect: 6 dummy variables included (not shown individually).

## 4. Discussion

This study examined the associations between nurse competencies, patient characteristics, and pain management protocol adherence, while accounting for between‐ICU clustering. The findings suggest that knowledge, self‐efficacy, and attitude are independently associated with protocol adherence. The full model explained 53.4% of the variance in adherence.

Recent integrative and scoping reviews published in 2026 have synthesized the available evidence linking ICU pain‐management competency with organizational context, highlighting the role of workload, staffing, resource availability, and educational opportunities as key contextual determinants [20, 21]. These reviews, however, remain primarily descriptive and call for empirical examination of the competency–adherence relationship within real‐world ICU settings. The present study responds to this gap by empirically testing the associations between nurse competencies, patient characteristics, and protocol adherence across seven ICUs, while accounting for between‐ICU clustering. Our findings extend the existing descriptive literature by quantifying the independent contributions of knowledge, self‐efficacy, and attitude to adherence and by demonstrating that patient‐level complexity—rather than between‐ICU clustering—explains a substantial portion of the variance in this sample.

Consistent with previous research [7, 8], knowledge was the strongest predictor of adherence (*β* = 0.311), followed by self‐efficacy (*β* = 0.290) and attitude (*β* = 0.276). The moderate levels of these competencies (59%–71% of maximum scores) align with findings from other ICU settings [22] and suggest significant room for improvement. The finding that only 36% of nurses had received formal pain management training is concerning and may explain the limited competency levels observed. Previous intervention studies have demonstrated that structured educational programs can significantly improve nurses’ knowledge, self‐efficacy, and attitudes toward pain management [23, 24].

The ICC of 0.02 (95% CI: 0.001–0.15) for protocol adherence across the seven ICUs indicates minimal clustering, suggesting that individual‐level factors are the primary correlates of adherence in this sample. This finding is consistent with implementation science frameworks emphasizing individual capability and motivation [25], but it also highlights the need for caution: With only seven ICU clusters, ICC estimates have wide confidence intervals, and the observed between‐ICU variability in adherence (range 7.6–8.9 out of 12) may not be fully captured. The organizational support composite ranged from 1 to 5 across the seven ICUs, and ICU‐level mean adherence ranged from 7.6 to 8.9. Given the small number of clusters (*n* = 7), these organizational findings are descriptive only and require confirmation in larger multilevel studies.

The persistent association of patient characteristics with adherence—even after adjusting for nurse competencies and ICU fixed effects—highlights the clinical complexity of pain management in certain patient populations. Patients with substance use history, communication inability, reduced consciousness, and mechanical ventilation consistently had lower adherence scores. This is clinically expected, as these patients require more complex assessment strategies, particularly validated behavioral tools for noncommunicative patients [26, 27]. The findings underscore the need for targeted training and organizational support for these challenging clinical situations [28].

The mean NAS across the seven ICUs was 81.6%, which is higher than the average reported in a recent systematic review [29]. However, because no inferential analysis of workload was conducted in this study, this observation is descriptive only and should be interpreted as hypothesis‐generating.

### 4.1. Implications for Nursing Management

Given the small number of ICU clusters (*n* = 7), the following implications are hypothesis‐generating rather than prescriptive. These findings suggest several potential implications for nurse managers. First, educational programs could be designed to be practical and competency‐based, incorporating simulation, case‐based learning, and supervised practice with feedback [23, 24]. Second, specific strategies may be needed for complex patient populations, including training in behavioral pain assessment tools and structured approaches for patients with substance use disorders [28]. Third, managers may consider conducting regular audits of protocol adherence with feedback [30, 31]. Future research should test these recommendations in formal multilevel studies with adequate cluster numbers before they are adopted as evidence‐based management guidelines.

### 4.2. Limitations

Several limitations warrant consideration. First, the cross‐sectional design precludes causal inferences. Second, self‐report competency measures may be subject to social desirability bias. Third, the study was conducted in one city in Iran, limiting generalizability. Fourth, organizational characteristics were measured at the ICU level with only seven clusters. Although ICU was entered as a fixed effect to adjust for clustering, this approach cannot fully substitute for formal multilevel modeling, and ICU‐level organizational support could not be included as an independent predictor because of collinearity with the ICU fixed effect. The ICC (0.02; 95% CI: 0.001–0.15) reflects all sources of between‐ICU variation and not organizational context alone. These findings are descriptive only and require replication in larger cluster samples using multilevel modeling. Fifth, unmeasured confounding variables may have influenced the observed relationships. Sixth, two components of the organizational support composite (documentation monitoring and analgesic/PCA pump availability) overlap conceptually with how the protocol adherence outcome was scored, which may have inflated the observed association. Seventh, inter‐rater reliability for the observational adherence checklist was not formally assessed, and the organizational support composite was constructed as an unweighted sum of three components without independent psychometric validation. Eighth, the internal consistency (Cronbach’s alpha) of the parent organizational questionnaire was not calculated because the questionnaire was completed at the ICU level with only seven units, which is insufficient for reliable reliability estimation. Ninth, the Hawthorne effect may have influenced nurse behavior during direct observation. Tenth, the rule for selecting the index patient (longest ICU stay when multiple eligible patients) may have introduced selection bias toward more complex patients.

## 5. Conclusions

This study provides evidence that nurse competencies and patient characteristics are independently associated with pain management protocol adherence in a multicenter ICU sample. Knowledge was the strongest predictor of adherence, followed by self‐efficacy and attitude. The ICC of 0.02 (95% CI: 0.001–0.15) indicates minimal between‐ICU clustering, suggesting that individual‐level factors are the primary correlates of adherence in this sample; however, this reflects all sources of between‐ICU variation and not organizational context alone. Given the small number of ICU clusters (*n* = 7) and the collinearity between organizational support and the ICU fixed effect, organizational findings are descriptive only and require confirmation in larger multilevel studies. Improving pain management may require a comprehensive approach that simultaneously addresses individual competency development and patient complexity [32].

## Author Contributions

Yasaman Shafie Motlagh Kashani: conceptualization, methodology, investigation, data curation, and writing–original draft. Mahboubeh Shali: conceptualization, methodology, supervision, and writing–review and editing. Maryam Esmaieli: validation, investigation, and writing–review and editing. Shima Haghani: formal analysis, statistical analysis, and writing–review and editing.

## Funding

This research received no specific grant from any funding agency in the public, commercial, or not‐for‐profit sectors.

## Disclosure

All authors approved the final version of the manuscript.

## Conflicts of Interest

The authors declare no conflicts of interest.

## Supporting Information

Additional supporting information can be found online in the Supporting Information section.

## Supporting information


**Supporting Information** Three supporting files accompany this manuscript. Supporting File 1 is the completed STROBE checklist for cross‐sectional studies, indicating the page on which each recommended item is reported. Supporting File 2 presents Table S1, which summarizes per‐ICU organizational characteristics (educational programs, resource availability, documentation monitoring, and the organizational support composite), nurse sample sizes, mean Nursing Activities Score, and protocol adherence values for the seven participating ICUs (525 nurses). Supporting File 3 contains the SPSS syntax used to reproduce the statistical analyses reported in the manuscript (SPSS Version 26).

## Data Availability

The datasets used and/or analyzed during the current study are available from the corresponding author on reasonable request.
