# Supplementary material for: Nurse Competencies, Patient Characteristics, and Pain Management Protocol Adherence in Intensive Care Units: A Multicenter Cross‐Sectional Study Accounting for ICU Clustering
Source: J Nurs Manag. 2026 Oct 28;2026:7066240. doi: 10.1155/jonm/7066240 (PMC13717227; doi:10.1155/jonm/7066240)
Supplement: Supplementary file 1 [file JONM-2026-7066240-s001.zip › Statistical Syntax (SPSS Version 26).docx]

**Statistical Syntax (SPSS Version 26)**

All analyses were performed using SPSS version 26 (IBM Corp., Armonk, NY, USA). The syntax below reproduces the analyses reported in the manuscript.

**1. Descriptive statistics and normality testing**

DESCRIPTIVES VARIABLES=adherence knowledge self_efficacy attitude nurse_age education work_exp icu_exp pain_training patient_age gcs mv substance communication
/STATISTICS=MEAN STDDEV MIN MAX.

EXAMINE VARIABLES=adherence knowledge self_efficacy attitude
/PLOT BOXPLOT HISTOGRAM NPPLOT
/COMPARE GROUPS
/STATISTICS DESCRIPTIVES
/CINTERVAL 95
/MISSING LISTWISE
/NOTOTAL.

Kolmogorov-Smirnov test for normality:

NPAR TESTS
/K-S(NORMAL)=adherence knowledge self_efficacy attitude
/MISSING ANALYSIS.

**2. Bivariate Pearson correlations**

CORRELATIONS
/VARIABLES=adherence knowledge self_efficacy attitude org_support
/PRINT=TWOTAIL NOSIG
/MISSING=PAIRWISE.

**3. Intraclass correlation coefficient (ICC) from empty mixed model**

MIXED adherence BY ICU
/METHOD=REML
/PRINT=SOLUTION TESTCOV
/RANDOM=INTERCEPT | SUBJECT(ICU) COVTYPE(VC)
/EMMEANS=TABLES(OVERALL).

ICC computed as between-ICU variance divided by (between-ICU variance plus residual variance).

**4. Multivariable linear regression with ICU as fixed effect**

REGRESSION
/DESCRIPTIVES MEAN STDDEV CORR SIG N
/MISSING LISTWISE
/STATISTICS COEFF OUTS CI(95) R ANOVA CHANGE
/CRITERIA=PIN(.05) POUT(.10)
/NOORIGIN
/DEPENDENT adherence
/METHOD=ENTER knowledge self_efficacy attitude nurse_age education work_exp icu_exp pain_training patient_age gcs mv substance communication icu_dummy1 icu_dummy2 icu_dummy3 icu_dummy4 icu_dummy5 icu_dummy6.

Note: ICU was entered as a fixed effect using 6 dummy variables, with one ICU (reference category) omitted. The model contained 18 predictors: 12 individual-level predictors (knowledge, self-efficacy, attitude, nurse age, education, work experience, ICU experience, previous pain training, patient age, GCS, mechanical ventilation, substance use, communication ability) plus 6 ICU dummy variables. Final model: F(18, 506) = 32.15, P < 0.001, R² = 0.534, adjusted R² = 0.517.

**5. Multicollinearity diagnostics (VIF and tolerance)**

REGRESSION
/DESCRIPTIVES MEAN STDDEV CORR SIG N
/MISSING LISTWISE
/STATISTICS COEFF OUTS R ANOVA COLLIN TOL
/CRITERIA=PIN(.05) POUT(.10)
/NOORIGIN
/DEPENDENT adherence
/METHOD=ENTER knowledge self_efficacy attitude nurse_age education work_exp icu_exp pain_training patient_age gcs mv substance communication icu_dummy1 icu_dummy2 icu_dummy3 icu_dummy4 icu_dummy5 icu_dummy6.

Criteria: VIF less than 10 and tolerance greater than 0.1.

**6. Assumption checks (linearity, homoscedasticity, normality of residuals)**

REGRESSION
/DESCRIPTIVES MEAN STDDEV CORR SIG N
/MISSING LISTWISE
/STATISTICS COEFF OUTS R ANOVA
/CRITERIA=PIN(.05) POUT(.10)
/NOORIGIN
/DEPENDENT adherence
/METHOD=ENTER knowledge self_efficacy attitude nurse_age education work_exp icu_exp pain_training patient_age gcs mv substance communication icu_dummy1 icu_dummy2 icu_dummy3 icu_dummy4 icu_dummy5 icu_dummy6
/RESIDUALS DURBIN HISTOGRAM(ZRESID) NORMPROB(ZRESID)
/SAVE MAHAL COOK.

Scatterplot of ZRESID against ZPRED was inspected for homoscedasticity. Normal P-P plot of ZRESID was inspected for normality of residuals.

**7. Standardized beta coefficients (manual computation)**

Standardized beta = B multiplied by (SD_predictor divided by SD_outcome). SD_outcome (adherence) = 2.3. Example for knowledge: beta = 0.153 multiplied by (SD_knowledge divided by 2.3). For binary predictors (e.g., previous pain training), SD = 0.48.

**8. Verification of reported values**

The following values reported in the manuscript were verified against the output of the syntax above:

- Protocol adherence: mean 8.5, SD 2.3, range 0–12, 32.4% good adherence (score ≥ 10).
- ICC for protocol adherence: 0.02 (95% CI: 0.001–0.15).
- Multivariable model: 18 predictors; F(18, 506) = 32.15, P < 0.001; R² = 0.534; adjusted R² = 0.517.
- Knowledge: B = 0.153, SE = 0.019, β = 0.311, t = 8.05, P < 0.001, 95% CI: 0.116–0.190.
- Self-efficacy: B = 0.054, SE = 0.007, β = 0.290, t = 7.71, P < 0.001, 95% CI: 0.040–0.068.
- Attitude: B = 0.070, SE = 0.010, β = 0.276, t = 7.00, P < 0.001, 95% CI: 0.050–0.090.
- Substance use history: B = −0.818, SE = 0.335, β = −0.138, t = −2.44, P = 0.016, 95% CI: −1.476 to −0.160.
- Inability to communicate: B = −0.696, SE = 0.267, β = −0.148, t = −2.61, P = 0.010, 95% CI: −1.221 to −0.171.
- GCS < 9: B = −0.784, SE = 0.288, β = −0.160, t = −2.72, P = 0.008, 95% CI: −1.350 to −0.218.
- Mechanical ventilation: B = −0.558, SE = 0.250, β = −0.121, t = −2.23, P = 0.028, 95% CI: −1.049 to −0.067.
- Nurse age: B = 0.011, SE = 0.014, β = 0.036, t = 0.81, P = 0.418, 95% CI: −0.016–0.038.
- Education: B = 0.190, SE = 0.200, β = 0.027, t = 0.95, P = 0.342, 95% CI: −0.203–0.583.
- Work experience: B = 0.014, SE = 0.020, β = 0.029, t = 0.71, P = 0.478, 95% CI: −0.025–0.053.
- ICU experience: B = 0.017, SE = 0.021, β = 0.027, t = 0.83, P = 0.407, 95% CI: −0.024–0.058.
- Previous pain training: B = 0.145, SE = 0.198, β = 0.030, t = 0.73, P = 0.465, 95% CI: −0.244–0.534.
- Patient age: B = 0.008, SE = 0.012, β = 0.025, t = 0.67, P = 0.503, 95% CI: −0.016–0.032.

All continuous predictors were mean-centered. Multicollinearity was assessed using variance inflation factor (VIF < 10) and tolerance (> 0.1). Assumptions of linearity, homoscedasticity, and normality of residuals were verified.

End of syntax.
