# Supplementary material for: Nurse Competencies, Patient Characteristics, and Pain Management Protocol Adherence in Intensive Care Units: A Multicenter Cross‐Sectional Study Accounting for ICU Clustering
Source: J Nurs Manag. 2026 Oct 28;2026:7066240. doi: 10.1155/jonm/7066240 (PMC13717227; doi:10.1155/jonm/7066240)
Supplement: Supplementary file 1 [file JONM-2026-7066240-s001.zip › Supplementary File 1.docx]

# STROBE (Supplementary File 1)

## STROBE Statement—Checklist of Items for Cross-Sectional Studies

**Manuscript Title:** ICU Organizational Support and Nurse Competencies in Pain Management Protocol Adherence: A Multicenter Cross-Sectional Study with Exploratory Cross-Level Moderation Analysis

**Authors:** Yasaman Shafie Motlagh Kashani, Mahboubeh Shali, Maryam Esmaieli, Shima Haghani

| **Section/Topic** | **Item No.** | **Recommendation** | **Page No.** | **Reported?** |
| --- | --- | --- | --- | --- |
| **Title and Abstract** | 1 | (a) Indicate the study's design with a commonly used term in the title or the abstract | 1 | ✅ Yes |
|  |  | (b) Provide in the abstract an informative and balanced summary of what was done and what was found | 3 | ✅ Yes |
| **Introduction** |  |  |  |  |
| Background/rationale | 2 | Explain the scientific background and rationale for the investigation being reported | 7-9 | ✅ Yes |
| Objectives | 3 | State specific objectives, including any prespecified hypotheses | 9 | ✅ Yes |
| **Methods** |  |  |  |  |
| Study design | 4 | Present key elements of study design early in the paper | 11 | ✅ Yes |
| Setting | 5 | Describe the setting, locations, and relevant dates, including periods of recruitment, exposure, follow-up, and data collection | 11 | ✅ Yes |
| Participants | 6 | (a) Give the eligibility criteria, and the sources and methods of selection of participants | 13-14 | ✅ Yes |
|  |  | (b) For matched studies, give matching criteria and number of exposed and unexposed | N/A | N/A |
| Variables | 7 | Clearly define all outcomes, exposures, predictors, potential confounders, and effect modifiers. Give diagnostic criteria, if applicable | 16-20 | ✅ Yes |
| Data sources/measurement | 8 | For each variable of interest, give sources of data and details of methods of assessment (measurement). Describe comparability of assessment methods if there is more than one group | 16-20 | ✅ Yes |
| Bias | 9 | Describe any efforts to address potential sources of bias | 25-26, 62-65 | ✅ Yes |
| Study size | 10 | Explain how the study size was arrived at | 13-14 | ✅ Yes |
| Quantitative variables | 11 | Explain how quantitative variables were handled in the analyses. If applicable, describe which groupings were chosen and why | 22-26 | ✅ Yes |
| Statistical methods | 12 | (a) Describe all statistical methods, including those used to control for confounding | 22-26 | ✅ Yes |
|  |  | (b) Describe any methods used to examine subgroups and interactions | 22-26 | ✅ Yes |
|  |  | (c) Explain how missing data were addressed | 13-14 | ✅ Yes |
|  |  | (d) If applicable, explain how loss to follow-up was addressed | 13-14 | ✅ Yes |
|  |  | (e) Describe any sensitivity analyses | 25-26 | ✅ Yes |
| **Results** |  |  |  |  |
| Participants | 13 | (a) Report numbers of individuals at each stage of study—e.g., numbers potentially eligible, examined for eligibility, confirmed eligible, included in the study, completing follow-up, and analyzed | 13-14, 27 | ✅ Yes |
|  |  | (b) Give reasons for non-participation at each stage | 13-14 | ✅ Yes |
|  |  | (c) Consider use of a flow diagram | N/A | N/A |
| Descriptive data | 14 | (a) Give characteristics of study participants (e.g., demographic, clinical, social) and information on exposures and potential confounders | 27-30 | ✅ Yes |
|  |  | (b) Indicate number of participants with missing data for each variable of interest | 13-14 | ✅ Yes |
| Outcome data | 15 | Report numbers of outcome events or summary measures | 31-33 | ✅ Yes |
| Main results | 16 | (a) Give unadjusted estimates and, if applicable, confounder-adjusted estimates and their precision (e.g., 95% confidence interval). Make clear which confounders were adjusted for and why they were included | 39-43 | ✅ Yes |
|  |  | (b) Report category boundaries when continuous variables were categorized | N/A | N/A |
|  |  | (c) If relevant, consider translating estimates of relative risk into absolute risk for a meaningful time period | N/A | N/A |
| Other analyses | 17 | Report other analyses done—e.g., analyses of subgroups and interactions, and sensitivity analyses | 45-51 | ✅ Yes |
| **Discussion** |  |  |  |  |
| Key results | 18 | Summarise key results with reference to study objectives | 53 | ✅ Yes |
| Limitations | 19 | Discuss limitations of the study, taking into account sources of potential bias or imprecision. Discuss both direction and magnitude of any potential bias | 62-65 | ✅ Yes |
| Interpretation | 20 | Give a cautious overall interpretation of results considering objectives, limitations, multiplicity of analyses, results from similar studies, and other relevant evidence | 53-58 | ✅ Yes |
| Generalisability | 21 | Discuss the generalisability (external validity) of the study results | 62-63 | ✅ Yes |
| **Other Information** |  |  |  |  |
| Funding | 22 | Give the source of funding and the role of the funders for the present study and, if applicable, for the original study on which the present article is based | 68 | ✅ Yes |

### Notes on STROBE Compliance

1. **Item 1(a):** The study design ("multicenter cross-sectional study") is indicated in the title and abstract.
2. **Item 1(b):** The abstract provides a balanced summary of aim, methods, results, and conclusion.
3. **Item 9 (Bias):** Potential sources of bias (self-report, social desirability, observational scoring) are discussed in the Limitations section.
4. **Item 12(c) (Missing data):** Nurses who completed less than 70% of questionnaires were excluded; no other missing data imputation was performed.
5. **Item 13(c) (Flow diagram):** Not applicable; no flow diagram is included as the study is a cross-sectional survey with a single data collection point.
6. **Item 16(b) (Category boundaries):** Continuous variables were not categorized; all were analyzed as continuous.
7. **Item 16(c) (Absolute risk):** Not applicable; the study examines associations, not risk prediction.
8. **Item 22 (Funding):** No specific funding was received.
