# Supplementary material for: Nurse Competencies, Patient Characteristics, and Pain Management Protocol Adherence in Intensive Care Units: A Multicenter Cross‐Sectional Study Accounting for ICU Clustering
Source: J Nurs Manag. 2026 Oct 28;2026:7066240. doi: 10.1155/jonm/7066240 (PMC13717227; doi:10.1155/jonm/7066240)
Supplement: Supplementary file 1 [file JONM-2026-7066240-s001.zip › Supplementary Table S1.docx]

**Supplementary Table S1. Per-ICU organizational characteristics, nurse sample sizes, and protocol adherence values (N = 7 ICUs; 525 nurses).**

| **ICU** | **Hospital** | **Nurse n** | **Adherence mean±SD** | **Org support** | **Educ (0–1)** | **Res (0–3)** | **Doc (0–1)** | **NAS (%)** |
| --- | --- | --- | --- | --- | --- | --- | --- | --- |
| 1 | Imam Khomeini | 85 | 8.9±2.1 | 5 | 1 | 3 | 1 | 95 |
| 2 | Valiasr | 80 | 8.7±2.2 | 4 | 1 | 2 | 1 | 88 |
| 3 | Cancer Institute | 70 | 8.5±2.3 | 3 | 0 | 2 | 1 | 80 |
| 4 | Ziaian | 75 | 8.4±2.4 | 3 | 0 | 3 | 0 | 82 |
| 5 | Baharloo | 72 | 8.2±2.3 | 2 | 0 | 2 | 0 | 76 |
| 6 | Shariati | 73 | 7.9±2.5 | 2 | 1 | 1 | 0 | 74 |
| 7 | Sina | 70 | 7.6±2.6 | 1 | 0 | 1 | 0 | 72 |
| Total / Weighted mean | — | 525 | 8.5±2.3 | 3.42±1.15 | — | — | — | 81.6±7.4 |

Notes: ICU = intensive care unit; NAS = Nursing Activities Score; Org support = organizational support composite (range 0–5); Educ = availability of educational programs (0–1); Res = resource availability (0–3); Doc = documentation monitoring (0–1). The organizational support composite was computed as the sum of educational programs (0–1), resource availability (0–3), and documentation monitoring (0–1), yielding a total range of 0–5. Adherence = pain management protocol adherence score (range 0–12). NAS is reported as the ICU-level mean value. The weighted mean for organizational support across the 525 nurses was 3.42 ± 1.15 (possible range 0–5; observed range 1–5). Because organizational support is an ICU-level variable (n = 7), no inferential P-value is reported.
